# Modeling and Analysis of Bio-Inspired, Reconfigurable, Piezo-Driven Vibration Isolator for Spacecraft

**DOI:** 10.3390/biomimetics9010029

**Published:** 2024-01-04

**Authors:** Yubo Zhang, Lintao Wang, Lin Li, Xiaoming Wang, Shuai He

**Affiliations:** 1 Chinese Academy of Sciences Key Laboratory of On-Orbit Manufacturing and Integration for Space Optics System, Changchun Institute of Optics, Fine Mechanics and Physics, Chinese Academy of Sciences, Changchun 130033, China; 2 University of Chinese Academy of Sciences, Beijing 100049, China; 3 Space Optoelectronic Measurement and Perception Lab, Beijing Institute of Control Engineering, Beijing 100190, China; 4 China Academy of Space Technology, Beijing 100094, China

**Keywords:** vibration isolation, bio-inspired, reconfigurable, inertial stability

## Abstract

The positioning accuracy of spacecraft in orbit is easily affected by low-frequency micro-vibrations of the environment and internal disturbances caused by the payload. Inspired by the neck structure of birds, this study devised a piezo-driven active vibration isolation unit with high stiffness. First, a dynamic model and two-sensor feedback control method for the isolation unit were developed, and the isolation mechanism and anti-disturbance characteristics were analyzed. Further, the stability of the closed-loop was verified. Simulation models of serial and parallel systems based on the proposed vibration isolation unit were implemented to demonstrate its feasibility. The results indicate that the proposed isolation units can provide excellent low-frequency vibration isolation performance and inertial stability and that they can effectively resist the internal disturbance of the payload. Moreover, its performance can be further improved via serial or parallel reconfiguration that facilitates its adaptation to the varied isolation requirements of spacecraft.

## 1. Introduction

Concurrent with advancements in aerospace technology, the accuracy requirements of spacecraft have become increasingly stringent, rendering them remarkably sensitive to external vibrations [1,2]. First, owing to the complex environment associated with spacecraft in orbit, low-frequency environmental vibration and residual vibration caused by position adjustment become the main factors limiting improvements in spacecraft inertial stability [3,4,5]. However, internal disturbances caused by the refrigeration machines, gyroscopes, and reaction flywheels inside the spacecraft must also be considered [5,6]. Therefore, an ideal vibration isolation system for spacecraft should have the following capabilities:

1. the ability to stabilize the payload in inertial space, effectively counteracting the low-frequency vibration transmitted by the base structure, and
2. the ability to resist the internal disturbance inside the payload to prevent vibration level deterioration.

Currently, the isolation techniques used in precision spacecraft can be categorized into passive and active vibration isolation techniques [7,8]. Passive isolators include springs, hyperelastic materials, nonlinear structures, and parallel mechanisms with positive and negative stiffnesses [9,10,11,12]. In addition, studies have been inspired by biological systems to design passive vibration isolators (using linear or nonlinear structures) that can achieve better isolation performances [13,14,15,16]. As a traditional isolation method, passive isolation has the advantages of good high-frequency isolation performance and no energy requirements, which are particularly important in aerospace applications [17]. However, in aerospace applications, to ensure the inertial stability of the payload, excellent low-frequency vibration isolation performances are required. Thus, the natural frequency and stiffness of passive systems should be as low as possible; this makes them easily susceptible to the internal disturbance of the payload. Moreover, amplification at the resonant frequency of the system cannot be suppressed solely using passive isolation technology [18].

Active vibration isolation technology is realized by introducing sensors and actuators into a passive system such that the aforementioned shortcomings can be addressed to a certain extent. Typical actuators include electromagnetic and piezoelectric actuators [19]. Given the advantages of electromagnetic actuators, such as high response bandwidth and high precision, many scholars have conducted extensive research in this regard [20]. Liu et al. designed an adaptive proportional controller that could eliminate 90% of the vibration energy at the resonance frequency [21]. Further, Liu et al. designed a dual-feedback controller based on proportional–integral–derivative (PID) control and robust control for active vibration isolation of spacecraft, which can reduce the displacement at the center of the payload by 70% [22]. Zhang et al. designed a data-driven feedforward control method that does not require an accurate plant model, and the vibration attenuation reaches −25 dB at 10 Hz [23]. To improve the vibration isolation performance at low frequencies, soft-mounted structures are usually used as passive support structures; therefore, they cannot resist the internal disturbance of the payload owing to their inherently low stiffness. To resolve this contradiction, a hard-mounted structure (rigid spring) combined with a feedforward control strategy has been adopted [24]. However, high-stiffness systems often require large forces to suppress low-frequency vibrations, and the performance of this scheme is limited by an electromagnetic actuator that cannot provide sufficient force. Beijen et al. adopted a soft-mounted structure combined with a feedforward controller to achieve considerable ability for low-frequency isolation. Further, the use of a closed-loop proximity sensor was proposed to increase the equivalent stiffness by 50 times and thus effectively improve the ability to resist disturbance. However, excessively large closed-loop gains would lead to robustness issues, and the system’s performance is limited by the resolution of the proximity sensor [25].

Piezoelectric actuators have the advantages of a large driving force, high resolution, fast response, and high stiffness [26]. As aforementioned, when there is an internal disturbance in the payload, the vibration level deteriorates; however, the isolation system based on piezoelectric actuators has sufficient anti-disturbance ability owing to its inherently high stiffness [27]. Wang et al. designed a controller that combined force feedback and adaptive control for earth observation spacecraft and resulted in an attenuation of −30 dB for the Stewart platform under periodic excitation [28]. Du et al. designed a proportional–differential controller for optical payloads in spacecraft, and the Stewart platform achieved an attenuation of −20 dB at the natural frequency of the system compared to the passive controller [29]. Song et al. adopted a composite controller combining feedforward and feedback control, which can achieve 30% attenuation of the excitation amplitude even under adverse conditions [30]. In their study on low-frequency vibration isolation, Hanieh et al. reduced the system’s angle frequency to 50% of that of the passive system through integral force feedback to achieve better vibration isolation performance [31].

**Figure 1:**
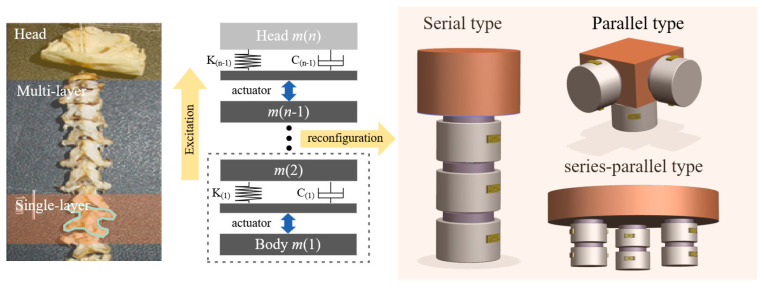
Architecture of a bio-inspired active vibration isolation unit.

Although there are many studies on active vibration isolation control, the balance problem between anti-disturbance ability and inertial stability remains to be satisfactorily addressed. This study draws inspiration from the phenomenon that birds can maintain their heads stable during movements; because birds cannot rotate their eyes over a wide range, they need to keep their heads inertially stable during movements to ensure that their field of vision remains unchanged [32,33,34]. As shown in Figure 1, the results of biological experiments on birds show that their necks comprise multiple sections in series, which have good rigidity and can effectively resist external disturbance, with each section implicating the next one by hinge and muscles. The skeletal muscles in each section act as an actuator, contracting when sensing the body shake to ensure the inertial stability of the head [35,36,37,38,39]. On the basis of the aforementioned biological research on bird necks, this paper proposes a bio-inspired space isolation unit. The architecture of the isolation unit is depicted in Figure 1, wherein each unit consists of an actuator in series with an elastic element that provides high-stiffness support (15–25 Hz) that can effectively resist disturbances from the payload. Piezoelectric actuators are used as actuators owing to their high stiffness and electrostrictive characteristics. They have sufficient driving force to eliminate low-frequency vibration from the foundation, thus overcoming the drawback of the poor low-frequency isolation performance of traditional high-stiffness isolators. As shown in Figure 1, each unit can be regarded as a single section in the neck with excellent low-frequency isolation ability to ensure payload stability in the inertial space while exhibiting high stiffness. In addition, inspired by the configuration of the bird neck, which is formed by multiple sections in series, this paper posits that a single isolation unit can be reconfigured in series or parallel to achieve better performance in satisfying the varied isolation requirements of spacecraft.

The remainder of this paper is organized as follows: Section 2 introduces the dynamic modeling of the vibration isolation unit and the design of the two-sensor feedback control algorithm. Section 3 presents an analysis of the stability of the system in accordance with the Bode diagram and root locus method. In Section 4, an electromechanical coupling simulation model is established to verify the effectiveness of the isolation unit, and reconfigurability is introduced. Section 5 summarizes the conclusions drawn from the study.

## 2. Active Isolation Unit

### 2.1. Dynamic Model

**Figure 2:**
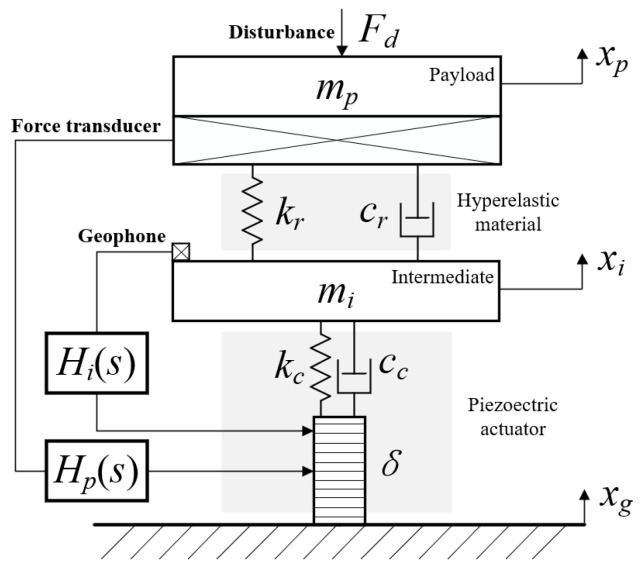
Dynamic model of the vibration isolation unit.

Inspired by the neck structure of birds, we designed a piezo-driven vibration isolation unit. Figure 2 illustrates the isolation unit, which consists of a piezoelectric actuator, intermediate mass, hyperelastic material, and payload. The sensor adopts a force sensor and geophone to simultaneously provide feedback. As an important passive isolation element, a hyperelastic material can effectively suppress the high-frequency modes of a system owing to its structural damping properties. In addition, to ensure that the isolation unit has sufficient ability to resist disturbance, the natural frequency should be maintained at 15–25 Hz (loaded state). Therefore, the shape and size of the hyperelastic material must be designed on the basis of simulations. To establish the damping force, the piezoelectric actuator adopts a viscous damping model [40,41], which is also used for hyperelastic materials to simplify the dynamic model. Furthermore, the nonlinear elastic characteristics of hyperelastic materials are not considered because of the small vibration magnitude of the spacecraft [18,42].

In Figure 2, *k_c_* and *c_c_* represent the stiffness and damping of the piezoelectric actuator; *m_i_* is the intermediate mass; *k_r_* and *c_r_* represent the stiffness and damping of the hyper-elastic material, respectively; and *m_p_* is the payload mass. When the base is excited, the displacement of the base is *x_g_*, the displacement of the intermediate mass is *x_i_*, the displacement of the payload is *x_p_*, the internal disturbance of the payload is *F_d_*, and the deflection of the piezoelectric actuator *δ*. The numerical values of these parameters are listed in Table 1.

### 2.2. Dynamic Analysis

The dynamic equation of the isolation unit is established as follows:

(1) $$m_{i}{\ddot{x}}_{i}=k_{c}\left(x_{g}-x_{i}+\delta \right)+c_{c}\left({\dot{x}}_{g}-{\dot{x}}_{i}+\dot{\delta }\right)-k_{r}\left(x_{i}-x_{p}\right)-c_{r}\left({\dot{x}}_{i}-{\dot{x}}_{p}\right)$$

(2) $$m_{p}{\ddot{x}}_{p}=k_{r}\left(x_{i}-x_{p}\right)+c_{r}\left({\dot{x}}_{i}-{\dot{x}}_{p}\right)$$

Owing to the small vibration magnitude of the spacecraft in orbit, the magnitude of the deflection of the piezoelectric actuator *δ* is on the order of micrometers. Further, as the proposed isolation unit mainly operates in the low-frequency region (<30 Hz), the first-order derivative of δ is also small. Furthermore, for lightly damped piezoelectric materials [40], the first-order derivative of *δ* should be ignored. The Laplace transform of (1) can be obtained as follows:

(3) $$\left(m_{i}s^{2}+c_{c}s+c_{r}s+k_{c}+k_{r}\right)x_{i}\left(s\right)=\left(c_{c}s+k_{c}\right)x_{g}\left(s\right)+k_{c}\delta \left(s\right)+\left(c_{r}s+k_{r}\right)x_{p}\left(s\right)$$

(4) $$\left(m_{p}s^{2}+c_{r}s+k_{r}\right)x_{p}\left(s\right)=\left(c_{r}s+k_{r}\right)x_{i}\left(s\right)$$

The displacement transfer function from base to intermediate mass can be derived as

(5) $$G_{1}\left(s\right)=\frac{x_{i}\left(s\right)}{x_{g}\left(s\right)}=\frac{m_{p}c_{c}s^{3}+\left(m_{p}k_{c}+c_{c}c_{r}\right)s^{2}+\left(c_{c}k_{r}+c_{r}k_{c}\right)s+k_{c}k_{r}}{m_{p}m_{i}s^{4}+As^{3}+Bs^{2}+Cs+k_{c}k_{r}}$$

$$\begin{array}{l} A=m_{p}c_{c}+m_{p}c_{r}+m_{i}c_{r}\\ B=m_{p}k_{c}+m_{p}k_{r}+m_{i}k_{r}+c_{r}c_{c}\\ C=c_{r}k_{c}+c_{c}k_{r} \end{array}$$

The displacement transfer function from the intermediate mass to the payload can be derived as follows:

(6) $$G_{2}\left(s\right)=\frac{x_{p}\left(s\right)}{x_{g}\left(s\right)}=\frac{c_{r}s+k_{r}}{m_{p}s^{2}+c_{r}s+k_{r}}$$

Evidently, the force sensor measurement is equal to the mass of the payload multiplied by its acceleration.

**Figure 3:**
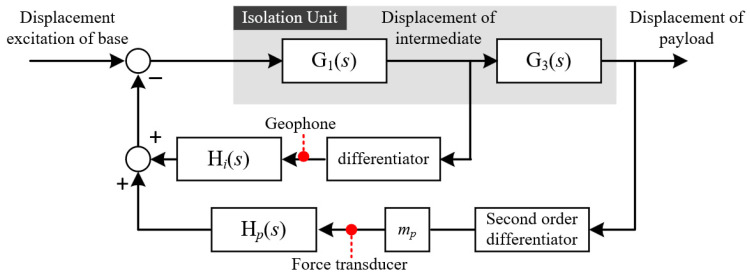
Control block diagram of isolation unit.

In summary, the displacement excitation of the foundation was transmitted to the load through the isolation unit, and the load was stabilized in the inertial space through the feedback control of the force sensors and geophones. A schematic of the piezoelectric isolation unit control system is shown in Figure 3.

### 2.3. Anti-Disturbance Characteristics

When the spacecraft is in orbit, the disturbance force of the internal components, such as the gyroscope and momentum wheel, deteriorates the payload vibration level. Therefore, the isolation unit for the spacecraft should have sufficient passive stiffness to resist disturbances. Considering the internal disturbance force *F_d_* of the payload, the dynamic equation of the isolation unit becomes

(7) $$\left(m_{i}s^{2}+c_{c}s+c_{r}s+k_{c}+k_{r}\right)x_{i}\left(s\right)=+k_{c}\delta \left(s\right)+\left(c_{r}s+k_{r}\right)x_{p}\left(s\right)$$

(8) $$\left(m_{p}s^{2}+c_{r}s+k_{r}\right)x_{p}\left(s\right)=\left(c_{r}s+k_{r}\right)x_{i}\left(s\right)+F_{d}\left(s\right)$$

The transfer function from the direct disturbance *F_d_* to the displacement of payload *x_p_* can be derived as

(9) $$\frac{x_{p}\left(s\right)}{F_{d}\left(s\right)}=\frac{m_{i}s^{2}+\left(k_{c}+c_{r}\right)s+k_{c}+k_{r}}{m_{p}m_{i}s^{4}+Ds^{3}+Es^{2}+Fs+k_{c}k_{r}}$$

$$\begin{array}{l} D=m_{p}c_{c}+m_{p}c_{r}+m_{i}c_{r}\\ E=m_{p}k_{c}+m_{p}k_{r}+m_{i}k_{r}+c_{r}c_{c}\\ F=c_{r}k_{c}+c_{c}k_{r} \end{array}$$

**Figure 4:**
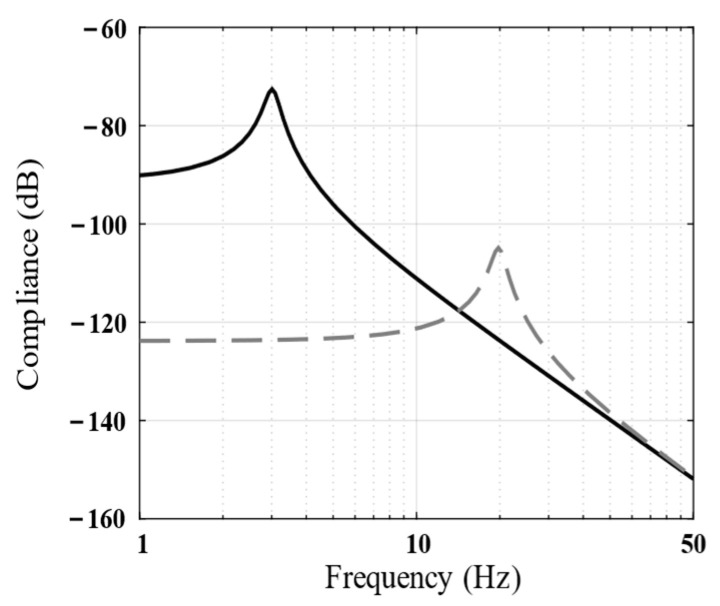
Bode magnitude plots of the compliance (analytical results) show the performance of the piezoelectric isolation unit (dotted line) and the commonly used soft-mounted isolation system (solid line).

The natural frequency of common soft-mounted isolation systems is 3–5 Hz [25]. Figure 4 shows the transfer function from the disturbance force to the payload displacement (compliance) of the piezoelectric isolation unit and soft-mounted isolation systems. As mentioned in Section 1, the ability of an isolation unit to resist disturbances largely depends on its stiffness, and the inherent stiffness of the proposed piezoelectric isolation unit is approximately 30 times that of the soft-mounted isolation unit.

### 2.4. Feedback Control

The closed-loop control of the piezoelectric isolation unit adopts two-sensor feedback that combines a force sensor and a geophone. First, the inertial displacement was obtained by integrating the absolute velocity signal of the geophone installed on the intermediate mass, and the inertial stability of the payload was ensured by increasing the feedback gain, similar to servo control. Second, a force sensor installed between the piezoelectric isolation unit and payload was used to suppress the system resonance peak caused by the hyperelastic element with integral force feedback. On the basis of the two-sensor feedback control loop, the extension and shortening of the piezoelectric actuator are controlled to realize the inertial stability of the payload; *δ* can be expressed as

(10) $$\delta =h_{i}{\dot{x}}_{i}+h_{p}F_{p}$$

Where *F_p_* is the signal measured by the force sensor, *h_i_* and *h_p_* are the controller corresponding to geophone and force sensor respectively. The piezoelectric actuator in the vibration isolation unit should have high precision and resolution. Therefore, a closed-loop displacement control method should be used to suppress the piezoelectric hysteresis effect, and the bandwidth of the piezoelectric actuator should be limited. In addition, to ensure the inertial stability of the payload, the frequency band that requires attention was primarily concentrated at low frequencies. Therefore, a low-pass filter *H_lp_* should be added to the feedback loop to limit the system bandwidth and reduce the high-frequency noise, with *ξ* = 0.7 and *ω_c_* = 50 × 2 pi rad/s.

(11) $$H_{lp}=\frac{\omega_{c}^{2}}{s^{2}+2\xi \omega_{c}s+\omega_{c}^{2}}$$

#### 2.4.1. Absolute Displacement Feedback

To improve the isolation ability of the piezoelectric isolation unit in the low-frequency range, the absolute displacement feedback was set to the integral of the absolute velocity of the intermediate mass so as to obtain its inertial displacement, and servo feedback control was adopted to ensure inertial stability by improving the feedback gain. The corresponding controller *h_i_* is expressed as follows:

(12) $$h_{i}=\frac{k_{i}}{s}$$

**Figure 5:**
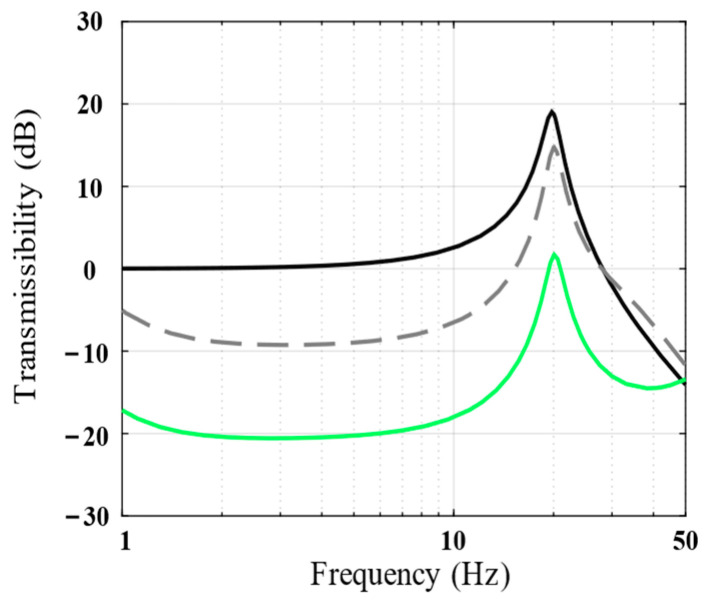
Bode magnitude plots of the vibration transmissibility (analytical results) show the performance of *k_i_* = 0 (black line), *k_i_* = 2 (dotted line), and *k_i_* = 10 (green line).

Figure 5 shows the vibration transmissibility of the piezoelectric isolation unit under different absolute displacement feedback gains, *k_i_*. As evident, the higher the *k_i_* value, the better would be the vibration isolation performance of the isolation unit in the low-frequency band.

#### 2.4.2. Integral Force Feedback

To further improve the performance of the isolation unit, the resonance peak in the low-frequency band must be suppressed. Therefore, a force sensor was placed between the isolation unit and payload, and sky-hook damping was realized by integrating the force feedback method. The controller *h_p_* is expressed as follows:

(13) $$h_{p}=\frac{k_{p}}{s}$$

**Figure 6:**
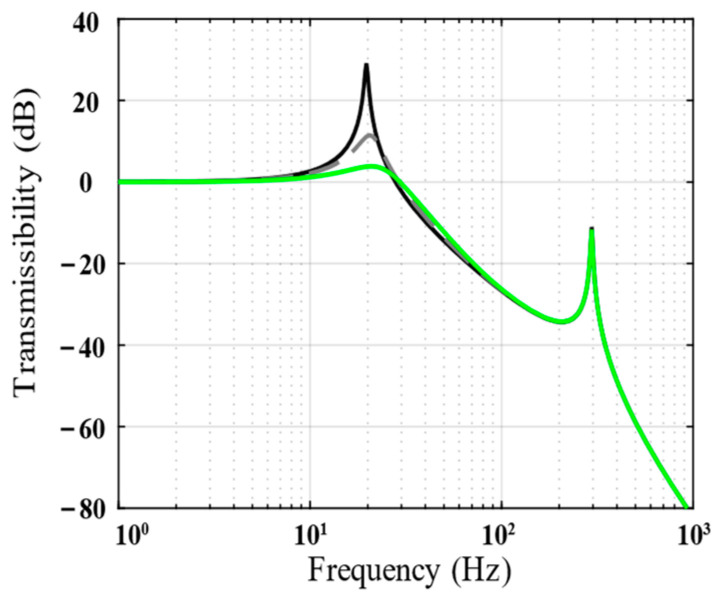
Bode magnitude plots of the vibration transmissibility (analytical results) show the performance of *k_p_* = 0 (solid line), *k_p_* = 0.002 (dotted line), and *k_p_* = 0.005 (green line).

Figure 6 shows the vibration transmissibility of the piezoelectric isolation unit under the passive state and integral force feedback. The integrated force feedback has a positive effect on suppressing the low-frequency resonance peak of the system.

Conversely, to avoid integral drift, a high-pass filter *H_hp_* also needs to be set in the closed loop, with *ξ* = 0.7 and *ω_i_* = 1 × 2 pi rad/s.

(14) $$H_{hp}=\frac{s^{2}}{s^{2}+2\xi \omega_{i}s+\omega_{i}^{2}}$$

In conclusion, the nominal controller *δ*(*s*) is as follows:

(15) $$\delta \left(s\right)={H_{hp}H}_{lp}k_{i}\frac{1}{s}x_{i}\left(s\right)+{H_{hp}H}_{lp}k_{p}\frac{1}{s}F_{p}\left(s\right)$$

## 3. Stability Analysis

### 3.1. Force Sensor Feedback Loop

**Figure 7:**
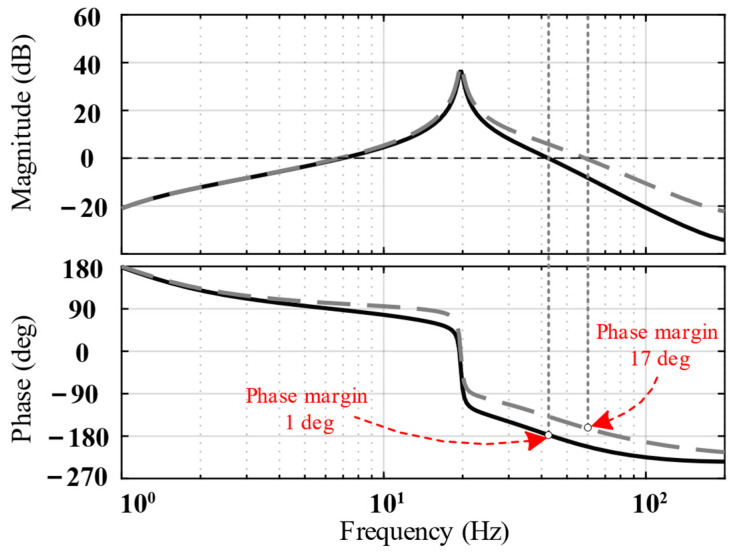
Open-loop Bode diagram of the system before compensation (solid line) and after compensation (dotted line).

The black curves in Figure 7 show an open-loop Bode diagram of the system. As shown, the phase margin was 1 degree, and the stability could not satisfy the requirements. Therefore, a lead compensator should be introduced at a suitable position to increase the phase margin and improve system stability.

Figure 7 shows that the crossing frequency of magnitude is approximately 45 Hz. To maximize the phase margin, it is necessary to ensure that the frequency of the compensator’s maximum phase is located at the crossing frequency of the compensated system. Therefore, the frequency of the compensator is set at 48 Hz, and the added lead compensator *G_c_*_1_(*s*) is as follows:

(16) $$G_{c1}\left(s\right)=\frac{a_{1}T_{1}s+1}{T_{1}s+1}$$

where *a*_1_ = 4.4 and *T*_1_ = 0.0016.

The Bode diagram after compensation is indicated using the gray curves in Figure 7. The phase margin after compensation increased to 17 degrees, indicating that the system’s stability improved.

### 3.2. Absolute Displacement Feedback Loop

**Figure 8:**
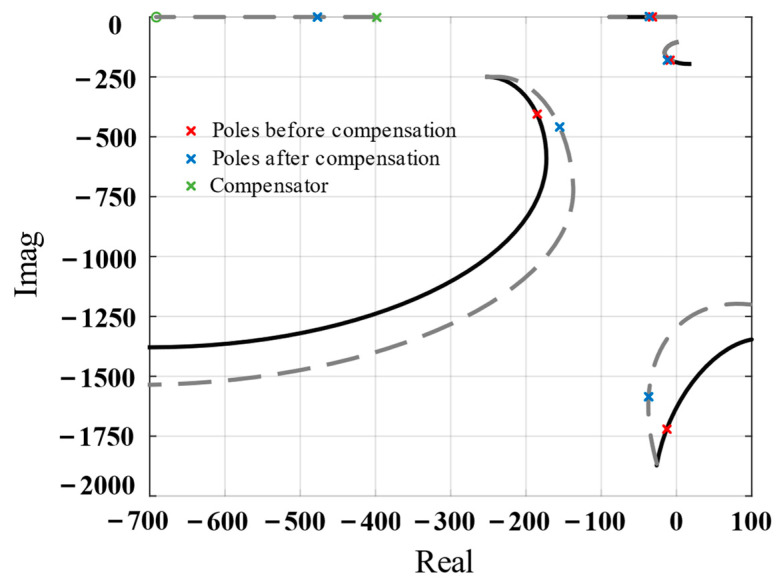
Root locus of the system before compensation (solid line) and after compensation (dotted line).

The black curve in Figure 8 shows the root locus diagram of the system, which indicates that the pole of the closed loop easily entered the right half of the S-plane. Therefore, compensation was required to improve the stability of the absolute displacement feedback loop. The added compensator *G_c_*_2_(*s*) is expressed as follows:

(17) $$G_{c2}\left(s\right)=\frac{a_{2}T_{2}s+1}{T_{2}s+1}$$

where *a*_2_ = 0.56 and *T*_2_ = 0.0025.

As shown in the root locus diagram of the compensated system, the pole prone to instability was pulled to the left. Further, it was difficult for the poles to enter the right half of the S-plane, indicating that the system’s stability improved.

In summary, the actual controller is

(18) $$\delta \left(s\right)={H_{hp}H}_{lp}k_{i}\frac{1}{s}x_{i}\left(s\right)G_{c2}\left(s\right)+{H_{hp}H}_{lp}k_{p}\frac{1}{s}F_{p}\left(s\right)G_{c1}\left(s\right)$$

**Figure 9:**
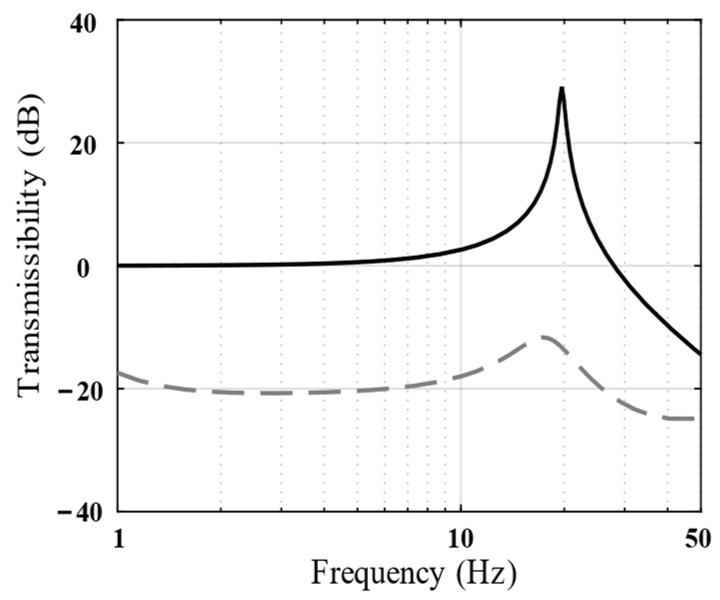
Bode magnitude plots of the vibration transmissibility (analytical results) show the performance of the passive system (solid line) and the active system (dotted line).

After the actual controller in Equation (18) is introduced into the closed loop, the vibration transmissibility of the piezoelectric isolation unit is shown in Figure 9. The vibration attenuation exceeds −15 dB in the 1–10 Hz range and exceeds −20 dB in the 20–50 Hz range; the isolator has excellent low-frequency isolation performance and can effectively improve the inertial stability of the payload.

## 4. Simulation

To examine the performance of the proposed piezoelectric isolation unit, we performed an electromechanical coupling simulation by using COMSOL Multiphysics finite element software combined with MATLAB/Simulink.

**Figure 10:**
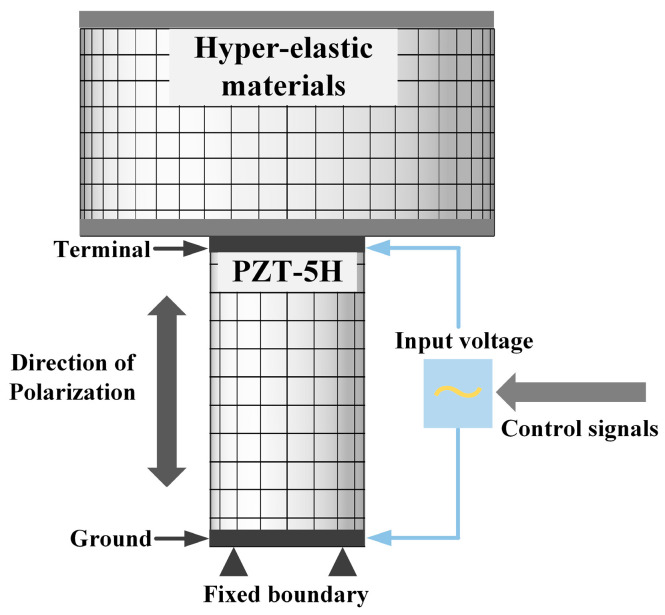
Finite element model of the isolation unit.

Figure 10 shows the finite element model of the vibration isolation unit. PZT−5H material with size Φ15 mm × 30 mm was selected for the piezoelectric actuator. The deformation of the actuator was provided by the electric field applied at both ends of the material, and the polarization direction of the piezoelectric material was the working direction of the isolation unit. The hyperelastic material was constructed using the two-parameter Mooney–Rivlin model with a size of Φ40 mm × 20 mm. The piezoelectric hysteresis effect must be considered to ensure the accurate modeling of piezoelectric materials. Therefore, to improve the positional accuracy of the piezoelectric actuator, an additional closed-loop displacement feedback control was built inside the actuator.

To comprehensively demonstrate the reconfigurability of the proposed piezoelectric isolation unit, typical serial and parallel systems based on the isolation unit were established in the simulation, and their performances were analyzed.

### 4.1. Serial System

**Figure 11:**
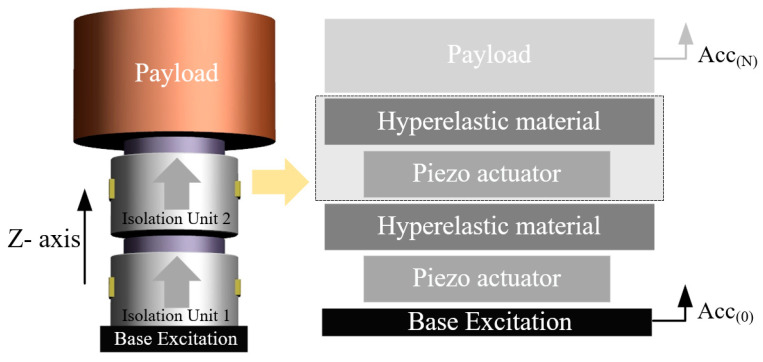
Configuration of the double-layer serial system.

To demonstrate the feasibility of the serial architecture of the proposed vibration isolation units, we constructed a double-layer serial system with two piezoelectric isolation units directly connected in series. The excitation from the base was successively transmitted to each vibration isolation unit, and each unit controlled the deflection of the actuators through feedback signals to ensure the inertial stability of the payload. Figure 11 shows the specific structure and mechanical model, and Table 2 lists the settings of the simulation parameters.

The natural frequency of the vibration isolation unit is 15 Hz, which can provide high-stiffness support and effectively resist disturbance forces inside the payload. In addition, because the mass of the isolation unit is considerably smaller than the payload mass, the coupling between the two isolation units can be ignored, and the parameters of each controller can be adjusted using the single-degree-of-freedom (DOF) analysis method described in Section 3.

**Figure 12:**
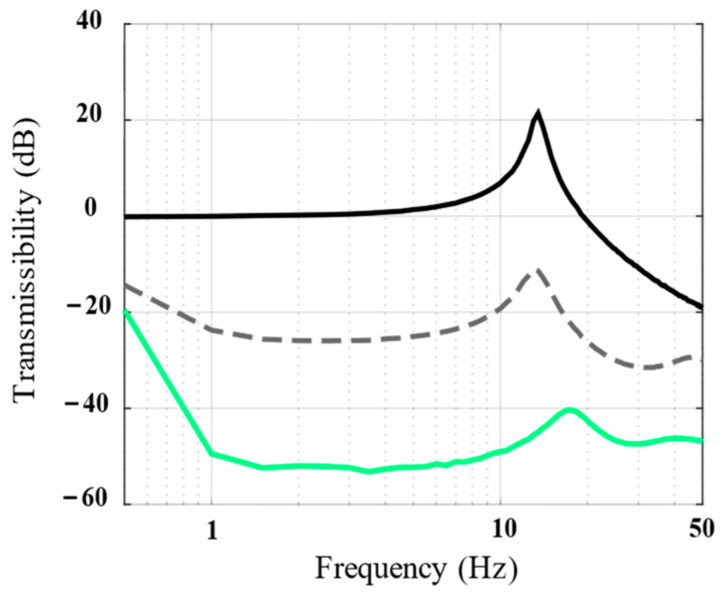
Bode magnitude plots of the vibration transmissibility (numerical results) show the performance of the passive system (solid line), single-layer unit (dotted line), and double-layer serial system (green line).

To illustrate the serial architecture, the vibration isolation performances of the passive state, single-layer isolation unit, and double-layer serial system were compared. The measured transfer function from the Z-direction acceleration of the base Acc(0) to the Z-direction acceleration response of the payload Acc(N) is shown in Figure 12 after applying white noise to the base. By observing the vibration transmissibility corresponding to the passive state and the single-layer isolation unit, we discovered that the single-layer isolation unit can attenuate the vibration from the base by −20 dB at 1–10 Hz; this is essentially consistent with the theoretical curve in Figure 9, demonstrating the effectiveness of the proposed piezoelectric vibration isolation unit. Furthermore, a comparison of the single-layer isolation unit with the double-layer serial system indicates that the low-frequency isolation performance can be further improved to −40 dB by using the double-layer serial architecture; this indicates that the proposed piezoelectric isolation unit has evident serial reconfigurability and that each individual unit can obtain better performance by simply stacking.

**Figure 13:**
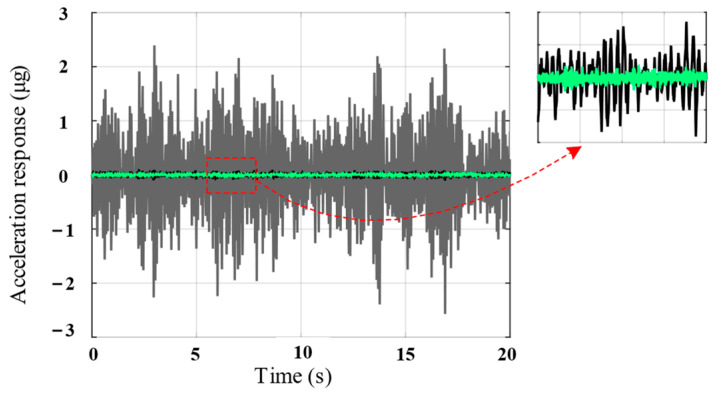
Acceleration response comparison of the passive system (gray line), single-layer unit (black line), and double-layer serial system (green line) under the same excitation (numerical results).

The time-domain curve of the payload acceleration response is shown in Figure 13. Compared with the passive state, the single-layer isolation unit can attenuate the amplitude of the acceleration response by approximately 30 times, whereas the double-layer serial system can attenuate it by approximately 5 times. Therefore, the inertial stability of the payload is significantly improved through a series combination of piezoelectric vibration isolation units.

The aforedescribed analysis indicates that the double-layer serial isolation system can significantly improve the inertial stability of the payload in the Z-direction. However, it does not work in other DOFs (DX, DY, RX, RY, and RZ). For spacecraft in orbit, vibration from the base has the characteristics of a wide frequency band and multiple DOFs, and serial architecture alone cannot satisfy the requirements of precision payloads. Thus, combining serial and parallel architectures is necessary to provide isolation with multiple DOFs.

### 4.2. Parallel System

**Figure 14:**
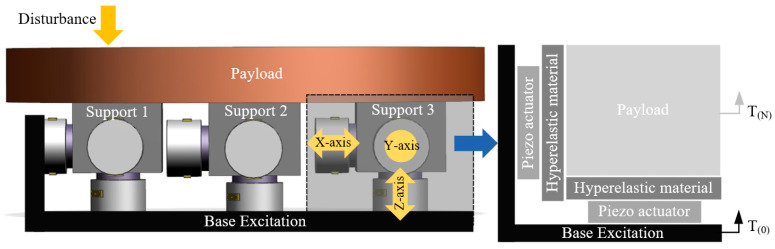
Configuration of the parallel isolation system.

Figure 14 shows the feasible parallel architecture of the piezoelectric isolation units. The payload was supported by three isolation components with a 120° distribution, and each component was composed of three piezoelectric isolation units distributed along the X-, Y-, and Z-axes of the global coordinate system. Excitation from the base was transmitted to the payload simultaneously through all nine isolation units, which worked together to ensure the inertial stability of the payload. In addition, the first six elastic modes of the system were higher than 15 Hz, which provided stable support for the payload. Table 3 lists the settings for the simulation parameters.

**Figure 15:**
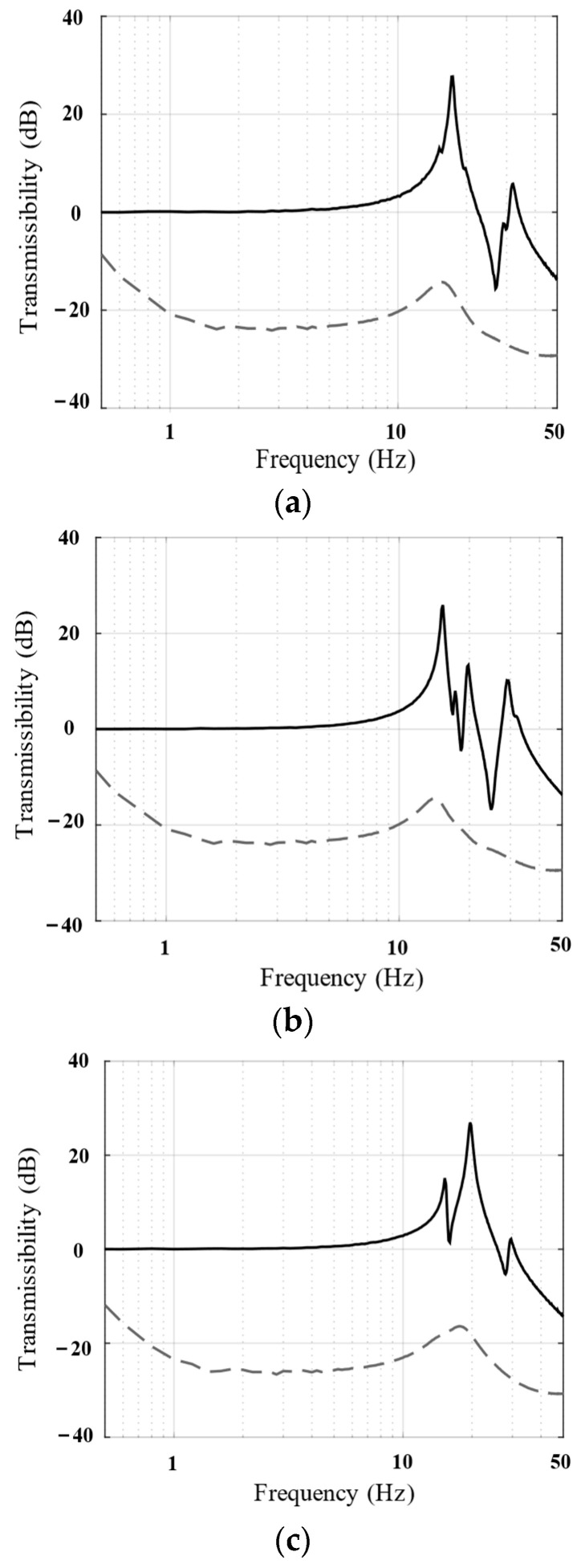
(**a**) X-axis; (**b**) Y-axis; (**c**) Z-axis. Bode magnitude plots of the vibration transmissibility (numerical results) show the performance of the passive system (solid line) and the active system (dotted line).

When spacecraft are excited by the base structure, the magnitude of the rotational vibration is usually much smaller than that of the translational vibration. Therefore, only the translational isolation performance of the system was analyzed. Figure 15 shows the vibration transmissibility in the X-, Y-, and Z-directions of the parallel isolation system. Evidently, the parallel architecture of the isolation unit provides excellent isolation performance with multiple DOFs and can provide attenuation of more than −20 dB in all three translational directions within 1–10 Hz, allowing the payload to maintain inertial stability over multiple DOFs.

**Figure 16:**
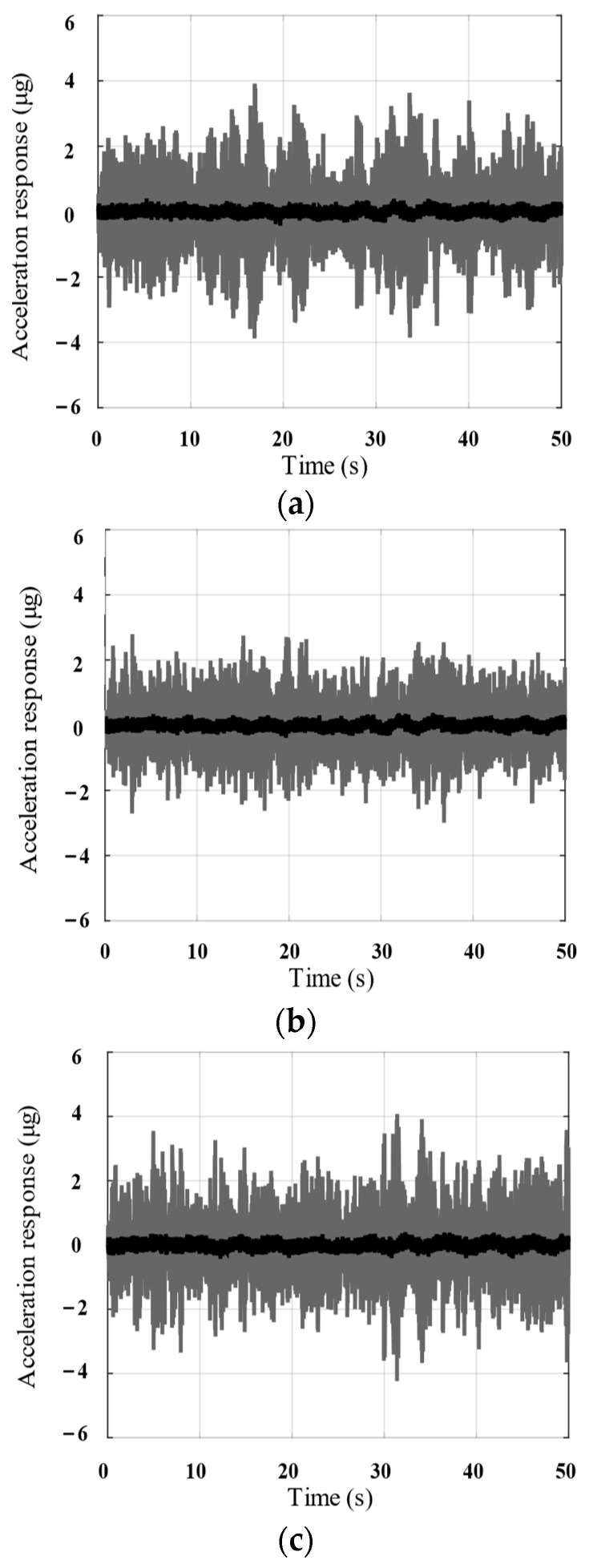
(**a**) X-axis; (**b**) Y-axis; (**c**) Z-axis. Acceleration response comparison of the active system (black line) and passive system (gray line) under the same excitation (numerical results).

In addition, as evident from the time-domain curve of the payload acceleration response in Figure 16, the parallel isolation system considered in this section can attenuate the amplitude of the acceleration response by more than 10 times in all three translational directions.

The aforedescribed analysis indicates that the proposed piezoelectric isolation unit has evident parallel reconfigurability, and the isolation performance with multiple DOFs can be obtained via parallel reconfiguration.

**Figure 17:**
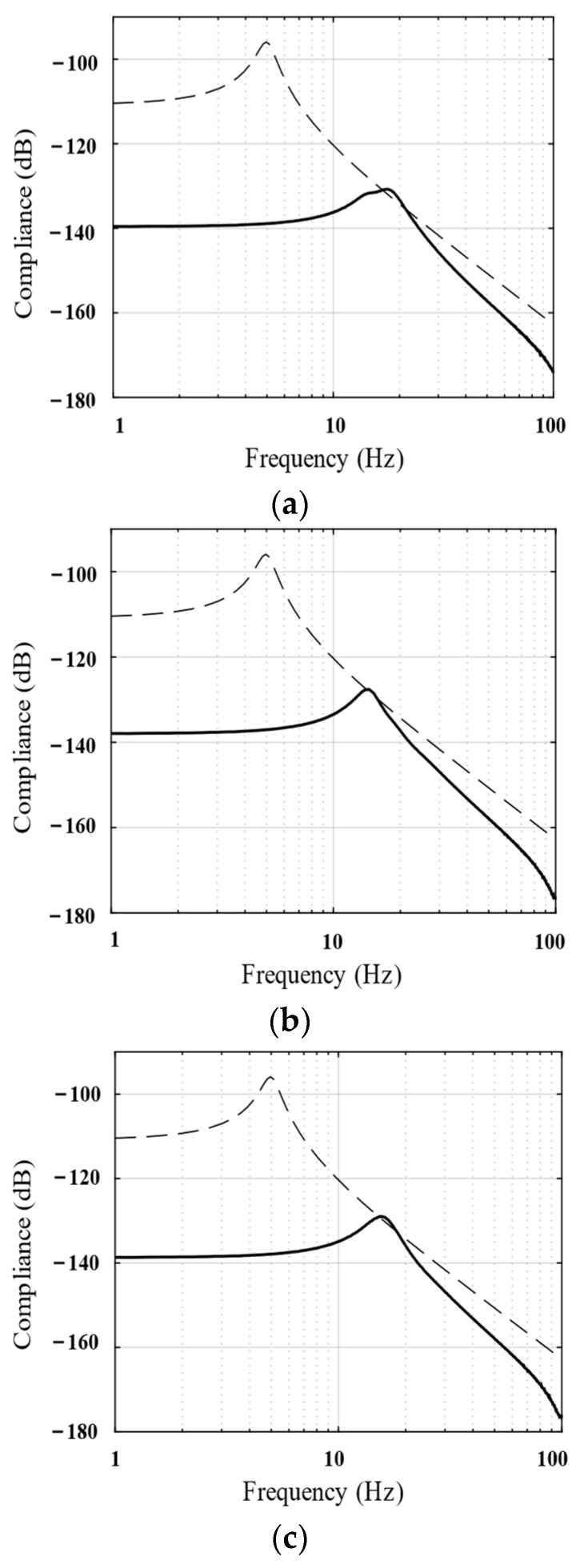
(**a**) X-axis; (**b**) Y-axis; (**c**) Z-axis. Bode magnitude plots of the compliance (numerical results) show the performance of the parallel isolation system (solid line) and the commonly used soft-mounted isolation unit (dotted line).

The aforementioned parallel isolation system can also resist possible disturbance forces in the payload owing to its inherently high stiffness. To illustrate these characteristics, the transfer function from the disturbance force to payload displacement (compliance) was measured in the simulation and compared with the common 3–5 Hz soft-mounted isolation system. The results are shown in Figure 17. The stiffness of the proposed vibration isolation system is approximately 30 times higher than that of the soft-mounted isolation system; this helps improve the overall stiffness and position accuracy of the spacecraft.

### 4.3. Discussion

The aforedescribed analysis shows that the proposed piezoelectric isolation unit has excellent low-frequency vibration isolation performance to ensure the inertial stability of the payload and sufficient stiffness to resist disturbances inside the payload. In addition, the analysis of serial and parallel systems based on the piezoelectric isolation unit revealed that the isolation unit can be reconfigured to expand its performance; in other words, the inertial stability can be improved through serial stacking, and the isolation capability of multiple DOFs can be obtained by parallel reconfiguration. Notably, the reconfiguration of the isolation unit is not limited to the examples considered in this study, and other forms of reconfiguration can be performed according to the actual isolation requirements of the spacecraft.

Furthermore, the energy consumption of the active isolators in spacecraft is a critical issue, and the energy consumption of the proposed isolator is closely related to the magnitude of vibration from the foundation and the mass of the payload; therefore, it has not been quantitatively analyzed in the simulation results. In fact, the proposed isolators may not be sufficiently friendly in terms of energy consumption, as the energy required to actively offset the low-frequency vibrations tends to be large, and energy harvesting technology based on the piezoelectric effect may solve this problem [43,44]. This issue is not the main focus of this study and will be quantitatively analyzed and meticulously considered in subsequent work and experiments.

## 5. Conclusions

Drawing inspiration from the phenomenon that birds can maintain their heads inertially stable when moving, an active vibration isolation unit for spacecraft was developed in this study. The main conclusions, based on theoretical analyses and simulation verification, are as follows:

First, an active isolation unit composed of a piezoelectric actuator and hyperelastic material was established, and its dynamic modeling and analysis were performed. Owing to its inherent high stiffness, the vibration isolation unit can effectively resist disturbances inside the spacecraft and prevent the deterioration of vibration levels due to disturbances.

Second, referring to the concept of servo control, a composite control method based on absolute displacement feedback and integral force feedback was proposed to improve the low-frequency isolation performance and inertial stability of the payload, and the stability of the control system was analyzed.

Finally, the isolation performance and anti-disturbance capability of the piezoelectric isolation unit were verified using an electromechanical coupling simulation model. A serial and parallel system based on an isolation unit was established, and it was found that the performance of the isolation unit could be extended by reconfiguration; that is, the inertial stability could be improved by serial stacking, and isolation capability with multiple DOFs could be obtained by parallel reconfiguration.

The proposed vibration isolation unit can address the spacecraft vibration problem caused by base excitation and internal disturbance and effectively maintain the inertial stability of the payload. On the basis of the reconfigurability of the isolation unit, the isolation system can be reconstructed according to the actual isolation requirements of the spacecraft, which has broad application prospects.

## Figures and Tables

**Table 1:** Numerical values of the parameters in the analytical model.

Parameter	Numerical Value
*m_p_*	200 kg
*k_r_*	3,400,000 N/m
*c_r_*	150 N/(m/s)
*m_i_*	30 kg
*k_c_*	100,000,000 N/m
*c_c_*	1500 N/(m/s)

**Table 2:** Numerical values of the parameters in the serial simulation system.

Parameter	Numerical Value
Mass of payload	196 kg
Z-axis stiffness of hyperelastic material	3,000,000 N/m
Z-axis damping of hyperelastic material	3000 N/(m/s)
Mass of intermediate	16 kg
Z-axis stiffness of the piezo actuator	80,000,000 N/m

**Table 3:** Numerical values of the parameters in the parallel simulation system.

Parameter	Numerical Value
Mass of payload	646 kg
single-axis stiffness of hyperelastic material	3,400,000 N/m
single-axis damping of hyperelastic material	1000 N/(m/s)
Mass of intermediate	27 kg
Z-axis stiffness of the piezo actuator	100,000,000 N/m

## Data Availability

The data that support the findings of this study are available from the corresponding author upon reasonable request.

## References

[1] Hu Q., Ma G. (2005). Variable Structure Control and Active Vibration Suppression of Flexible Spacecraft during Attitude Maneuver. Aerosp. Sci. Technol..

[2] Liu Y., Fu Y., He W., Hui Q. (2018). Modeling and Observer-Based Vibration Control of a Flexible Spacecraft with External Disturbances. IEEE Trans. Ind. Electron..

[3] Yang K., Zhang Y.-W., Ding H., Yang T.-Z., Li Y., Chen L.-Q. (2017). Nonlinear Energy Sink for Whole-Spacecraft Vibration Reduction. J. Vib. Acoust..

[4] Connolly C. (2009). Vibration Isolation Theory and Practice. Assem. Autom..

[5] Di Gennaro S. (2003). Output Stabilization of Flexible Spacecraft with Active Vibration Suppression. IEEE Trans. Aerosp. Electron. Syst..

[6] Zhang Y., Li M., Zhang J. (2017). Vibration Control for Rapid Attitude Stabilization of Spacecraft. IEEE Trans. Aerosp. Electron. Syst..

[7] Lee J.H., Kim H.Y., Kim K.H., Kim M.H., Lee S.W. (2017). Control of a Hybrid Active-Passive Vibration Isolation System. J. Mech. Sci. Technol..

[8] Abhilash M., Rajendran D., Sharma G. Passive Flexural Ring Micro-Vibration Isolator for Spacecraft Actuators. Proceedings of the 11th National Conference and Exhibition on Aerospace & Defense Related Mechanisms.

[9] Balaji P.S., Rahman M.E., Moussa L., Lau H.H. (2015). Wire Rope Isolators for Vibration Isolation of Equipment and Structures–A Review. IOP Conference Series: Materials Science and Engineering, Proceedings of the 9th Curtin University of Technology Science and Engineering International Conference 2014 (CUTSE2014), Sarawak, Malaysia, 3–4 December 2014.

[10] Lai A., Du Z., Gan C.L., Schuh C.A. (2013). Shape Memory and Superelastic Ceramics at Small Scales. Science.

[11] Kovacic I., Brennan M.J., Waters T.P. (2008). A Study of a Nonlinear Vibration Isolator with a Quasi-Zero Stiffness Characteristic. J. Sound Vib..

[12] Asadi Jafari M.H., Zarastvand M., Zhou J. (2023). Doubly Curved Truss Core Composite Shell System for Broadband Diffuse Acoustic Insulation. J. Vib. Control.

[13] Wu Z., Jing X., Bian J., Li F., Allen R. (2015). Vibration Isolation by Exploring Bio-Inspired Structural Nonlinearity. Bioinspir. Biomim..

[14] Gatti G., Ledezma-Ramirez D.F., Brennan M.J. (2023). Performance of a Shock Isolator Inspired by Skeletal Muscles. Int. J. Mech. Sci..

[15] Dai H., Jing X., Wang Y., Yue X., Yuan J. (2018). Post-Capture Vibration Suppression of Spacecraft via a Bio-Inspired Isolation System. Mech. Syst. Signal Process..

[16] Yan G., Wang S., Zou H., Zhao L., Gao Q., Zhang W. (2020). Bio-Inspired Polygonal Skeleton Structure for Vibration Isolation: Design, Modelling, and Experiment. Sci. China Technol. Sci..

[17] Jiao X., Zhang J., Li W., Wang Y., Ma W., Zhao Y. (2023). Advances in Spacecraft Micro-Vibration Suppression Methods. Prog. Aerosp. Sci..

[18] Liu C., Jing X., Daley S., Li F. (2015). Recent Advances in Micro-Vibration Isolation. Mech. Syst. Signal Process..

[19] Fuller C.R., von Flotow A.H. (1995). Active Control of Sound and Vibration. IEEE Control Syst. Mag..

[20] Shang J., Tian Y., Li Z., Wang F., Cai K. (2015). A Novel Voice Coil Motor-Driven Compliant Micropositioning Stage Based on Flexure Mechanism. Rev. Sci. Instrum..

[21] Liu Y.-H., Hsieh H.-E., Wu W.-H., Liao W.-H. (2014). An Active Vibration Isolation System Using Adaptive Proportional Control Method.

[22] Liu J., Li Y., Zhang Y., Gao Q., Zuo B. (2014). Dynamics and Control of a Parallel Mechanism for Active Vibration Isolation in Space Station. Nonlinear Dyn..

[23] Zhang Z., Yin W. Data-Driven Feedforward Control on Active Vibration Isolation System. Proceedings of the 2017 17th International Conference on Control, Automation and Systems (ICCAS).

[24] Beijen M.A., Heertjes M.F., Van Dijk J., Hakvoort W.B.J. (2018). Self-Tuning MIMO Disturbance Feedforward Control for Active Hard-Mounted Vibration Isolators. Control Eng. Pract..

[25] Beijen M.A., Heertjes M.F., Butler H., Steinbuch M. (2019). Mixed Feedback and Feedforward Control Design for Multi-Axis Vibration Isolation Systems. Mechatronics.

[26] Sui L., Xiong X., Shi G. (2012). Piezoelectric Actuator Design and Application on Active Vibration Control. Phys. Procedia.

[27] Li P., Fu J., Wang Y., Xing Z., Yu M. Dynamic Model and Parameters Identification of Piezoelectric Stack Actuators. Proceedings of the 26th Chinese Control and Decision Conference (2014 CCDC).

[28] Wang C., Xie X., Chen Y., Zhang Z. (2016). Investigation on Active Vibration Isolation of a Stewart Platform with Piezoelectric Actuators. J. Sound Vib..

[29] Du L., Ji L., Luo Y., Shao S., Xu M. Simulation and Experiment of an Active-Passive Isolator for Micro-Vibration Control of Spacecraft. Proceedings of the 2020 15th Symposium on Piezoelectrcity, Acoustic Waves and Device Applications (SPAWDA).

[30] Song H., Shan X., Hou W., Wang C., Sun K., Xie T. (2023). A Novel Piezoelectric-Based Active-Passive Vibration Isolator for Low-Frequency Vibration System and Experimental Analysis of Vibration Isolation Performance. Energy.

[31] Hanieh A.A., Preumont A. (2011). Multi-Axis Vibration Isolation Using Different Active Techniques of Frequency Reduction. J. Vib. Control.

[32] Sun X., Wang F., Xu J. (2021). A Novel Dynamic Stabilization and Vibration Isolation Structure Inspired by the Role of Avian Neck. Int. J. Mech. Sci..

[33] Chin D.D., Lentink D. (2016). Flapping Wing Aerodynamics: From Insects to Vertebrates. J. Exp. Biol..

[34] Deng T., Wen G., Ding H., Lu Z.-Q., Chen L.-Q. (2020). A Bio-Inspired Isolator Based on Characteristics of Quasi-Zero Stiffness and Bird Multi-Layer Neck. Mech. Syst. Signal Process..

[35] Shyy W., Aono H., Kang C., Liu H. (2013). An Introduction to Flapping Wing Aerodynamics.

[36] Mazaheri K., Ebrahimi A. (2011). Experimental Investigation on Aerodynamic Performance of a Flapping Wing Vehicle in Forward Flight. J. Fluids Struct..

[37] McArthur K.L., Dickman J.D. (2011). State-Dependent Sensorimotor Processing: Gaze and Posture Stability during Simulated Flight in Birds. J. Neurophysiol..

[38] Kress D., Van Bokhorst E., Lentink D. (2015). How Lovebirds Maneuver Rapidly Using Super-Fast Head Saccades and Image Feature Stabilization. PLoS ONE.

[39] Hedenström A., Johansson L.C. (2015). Bat Flight: Aerodynamics, Kinematics and Flight Morphology. J. Exp. Biol..

[40] Gu G.-Y., Zhu L.-M., Su C.-Y., Ding H., Fatikow S. (2014). Modeling and Control of Piezo-Actuated Nanopositioning Stages: A Survey. IEEE Trans. Autom. Sci. Eng..

[41] Adriaens H., De Koning W.L., Banning R. (2000). Modeling Piezoelectric Actuators. IEEEASME Trans. Mechatron..

[42] Li L., Yu Y., Wang L., Yuan L., Zhang L., Gong X., Wu Y., Zheng R. (2023). Modeling and Analysis of the Influence Caused by Micro-Vibration on Satellite Attitude Control System. Acta Astronaut..

[43] Zhang Y., Yang T., Du H., Zhou S. (2023). Wideband Vibration Isolation and Energy Harvesting Based on a Coupled Piezoelectric-Electromagnetic Structure. Mech. Syst. Signal Process..

[44] Zhou S., Lallart M., Erturk A. (2022). Multistable Vibration Energy Harvesters: Principle, Progress, and Perspectives. J. Sound Vib..

